# Real-world evidence on data quality in precision oncology platforms: insights from the Molecular Twin Research Umbrella protocol

**DOI:** 10.3389/fdgth.2026.1828544

**Published:** 2026-06-30

**Authors:** Michael Zuniga, Denis Marino, Yuan Yuan, Jin Sun Lee, Nazelee Dagliyan, Dominique Pope, Gangothri Namasivayam, Hui Hong, Grant Dagliyan, Warren G. Tourtellotte, Robert Figlin, Karine Sargsyan

**Affiliations:** 1 OncoBiobank Shared Resource, Cancer Center, Cedars-Sinai Medical Center, Los Angeles, CA, United States; 2 Samuel Oschin Comprehensive Cancer Center, Cedars-Sinai Medical Center, Los Angeles, CA, United States; 3 Department of Pathology and Laboratory Medicine, Neurology, Neurosurgery and Biomedical Sciences, Cedars-Sinai Cancer Center, Los Angeles, CA, United States; 4 F. Widjaja Inflammatory Bowel Institute, Cedars-Sinai MIRIAD IBD Biobank, Los Angeles, CA, United States; 5 International Biobanking and Education, Medical University of Graz, Graz, Austria; 6 Department of Digital Medicine and Artificial Intelligence, Yerevan State Medical University, Yeveran, Armenia

**Keywords:** biobanking infrastructure, biospecimen corresponding data quality, data harmonization, data quality and governance, electronic health records (EHR), longitudinal cancer cohorts, longitudinal study, precision medicine

## Abstract

The increase in precision oncology largely relies on the availability of high-quality, longitudinal, and multi-source, multi-purpose, and multidimensional corresponding data that integrate clinical, pathological, and molecular information. While new and advanced biomedical methods enable large-scale data generation, the operational challenges associated with data retrieval, harmonization, and quality control remain insufficiently described in scientific literature. In parallel, data incompleteness and heterogeneity in collection practices and coding standards are diminishing confidence in precision medicine programs. This methodological case study focused on the challenges of data harmonization within the Molecular Twin Research Umbrella Protocol at Cedars-Sinai Medical Center. This systematic four-stage process includes requirements for data access and assembly, processing for cleaning and harmonization, and quality verification before distribution. Pairwise comparisons have been made for each type of record, from electronic health records through cancer registration and biobanking. The demographics showed a very high concordance (>95%), whereas the clinically essential variables, such as tumor TNM stage, diagnostic specificity, and intervention schedules, showed moderate discordance (14.8%–17%). These discrepancies are a significant hindrance to the readiness of the cohorts for prediction models. Based on these results, we propose mitigation strategies aimed at improving the accuracy, completeness, and standardization of longitudinal oncology cohort datasets. This study provides key recommendations for long-term oncology cohorts and for the development of digital twin infrastructure for any institution, stressing that sound data quality infrastructure is a cornerstone of trustworthy precision oncology and translational research.

## 1 Introduction

Precision medicine represents a revolution in oncology treatment by enabling the development of patient-specific strategies for the prevention, diagnosis, and treatment of disease through the integration of genomic, clinical, environmental, and lifestyle factors (1–3). Advances in high-throughput sequencing and multi-omics technologies have enabled the characterization of tumor and host response at the molecular level, supporting the establishment of precision oncology platforms that leverage multi-omics and clinical information to determine a patient's treatment course (4). However, despite the rapid proliferation of such platforms, concerns have been raised regarding the quality of the accompanying clinical and omics data across datasets generated at different medical institutions (5).

Longitudinal cancer cohorts represent a unique resource, serving as models for the temporal development of cancer and resistance. In this context, biobanking within healthcare systems is a vital instrument for enabling longitudinal cohort collection, allowing for comprehensive clinical data analysis (6). Despite considerable commitment to mobile health (mHealth) technology development and implementation, healthcare information remains fragmented and stored across various systems, including Electronic Health Records (EHRs), tumor/cancer registration systems, and Laboratory Information Management Systems (LIMS) (7–11). The variability in documentation practices, coding schemes, updates, and models has implications for cohort assembly and analysis. Incomplete and inconsistent data on stage and therapies in cancer cases have been recognized as affecting the quality of predictive models and survival analyses (12–17). Moreover, the issue is even more apparent in the context of precision oncology tools for analyzing and constructing digital twins for artificial intelligence (18).

High-quality data provenance and lineage are essential for constructing comprehensive and credible datasets. Data provenance describes the origins of the data and its subsequent processing, whereas data lineage tracks relationships among different data items—for instance, which omics datasets were queried to infer a response or resistance to a specific therapy (19, 20). The lack of a robust provenance-tracking mechanism may significantly restrict the utility of large, shared, or federated datasets for downstream analyses, hinder reproducibility, and increase the risk of biases.

The traceability of timestamps for data input is also vital for ensuring timeliness in building and interpreting models. Timestamps documenting a patient's understanding of their tumor profile and the implementation of the corresponding treatment are integral to reconstructing a molecular burden of therapy and response chain (9).

The response to therapy in cancer is known to depend on various factors, such as the timing of treatment initiation relative to tumor profiling and the time elapsed since surgery or neoadjuvant therapy before adjuvant therapy commencement. Thus, integrating timestamps from multiple data sources enhances data quality and enables the inference of additional therapeutic opportunities. Comprehensive data provenance tracking and lineage generation also play a vital role in ensuring the accuracy, interpretability, and flexibility of models and decision-making processes that combine human expertise with artificial intelligence (AI). This is especially important for our internal collections where the patient-specific molecular twin will be developed from data gathered within the Molecular Twin Research Umbrella Protocol.

The Molecular Twin Study Umbrella Protocol at Cedars-Sinai Medical Center aimed to facilitate the combined analysis of biospecimens and longitudinal clinical data across various cancers (7, 8). While the scientific applications of molecular and digital twins are growing, there is comparatively less research focusing on data infrastructure operations that support such platforms. In this manuscript, we present a case study of the challenges, pitfalls, and mechanisms for overcoming longitudinal oncology data harmonization in an academic hospital setting. This is more of an infrastructure-level experience-sharing effort with implications for precision oncology and digital medicine initiatives worldwide, and it is not a hypothesis-based study.

## 2 Materials and methods

### 2.1 Study governance and ethics

This analysis was completed under the Molecular Twin Study Umbrella Protocol (prospective-biobanking type protocol) approved by the Cedars-Sinai Institutional Review Board (STUDY00001879). This protocol allows for the consented collection and integration of biospecimens and associated clinical, pathological, and molecular data across various cancer types. So far, over 3,200 participants have been recruited across different cancer types.

The protocol enables both prospective collection of biospecimens and retrospective and prospective clinical data access related to cancer diagnosis and treatment, helping to reconstruct patient trajectories throughout the course of disease. Biospecimens include tissue specimens and blood samples collected during standard clinical care. Longitudinal blood collection is performed twice a year during follow-up visits or during significant clinical events, such as disease progression or therapy changes.

### 2.2 Cohort scope and use cases

Evaluation of data recovery and harmonization procedures was conducted for several requests for breast cancer cohorts initiated by researchers. The use cases included: (i) breast cancer (stage I–III) with frequent blood draws post-resection and surveillance; (ii) triple-negative breast cancer with pre-immune checkpoint blockage (ICB) pathology; and (iii) antibody-drug conjugate therapy with multiple blood collection timepoints (pre-, during-, post-immunotherapy). The use cases population comprised several hundred patients enrolled and followed longitudinally between June 2022 to June 2025, with follow-up duration varying depending on clinical trajectory and data availability.

### 2.3 Data sources

**Figure 1:**
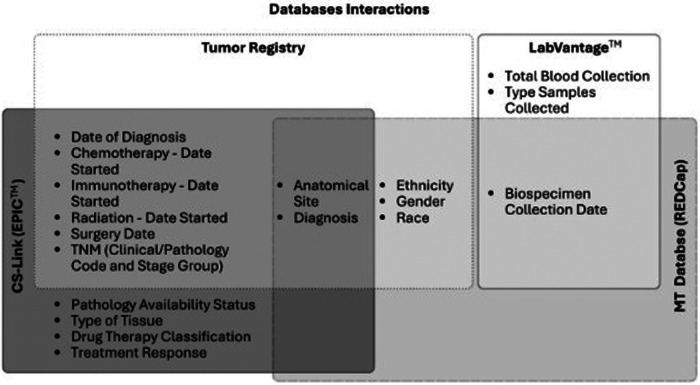
Comprehensive schematic of data management from request to dataset release in the molecular twin umbrella protocol.

Data were retrieved from multiple institutional databases, manually extracted from the institutional electronic health record (CS-Link, EPIC™), and reports were gathered from tumor registry, the laboratory information management system (LabVantage™), and Molecular Twin database built in REDCap software. Each system was developed independently for distinct operational purposes with a different update strategy and data structure. Figure 1 shows the variability in design and purpose of each system and the variables extracted from the sources, included demographic information (e.g., age, sex, race, ethnicity), tumor characteristics (e.g., diagnosis, date of diagnosis, histology, TNM code and stage group, genetic test availability), treatment-related variables (e.g., type and timing of interventions, drug therapy classification, treatment responses), and biospecimen data (e.g., sample type, collection timepoints, and availability). All variables assessed are presented in the Supplementary Table S1.

### 2.4 Data management workflow

**Figure 2:**
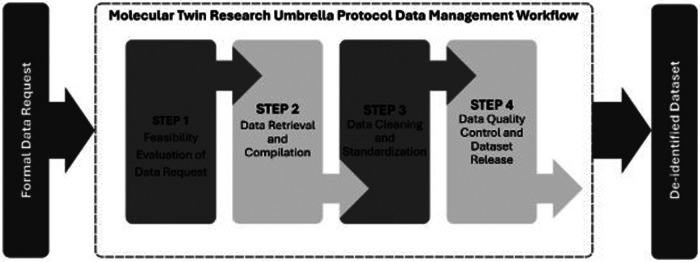
Workflow of data management process established to address data requests under 1 the molecular twin research umbrella protocol.

Processing data requests from investigators consisted of a four-step standardized process: data request entry and evaluation for feasibility, data compilation from various systems, data cleansing and conversion to a standardized format, quality checks, and distribution of data (Figure 2). All requests for access to the data are subject to institutional reviews.

#### 2.4.1 Feasibility evaluation of data request and data retrieval and compilation

All data requests were reviewed to assess feasibility based on data availability and alignment with protocol and institutional requirements. Once approved, data retrieval and compilation were initiated using the Molecular Twin database as the primary source, complemented by clinical data manually extracted from the CS-Link electronic health record system and compared with data from tumor registries. The compiled dataset was then prepared for subsequent data cleaning, harmonization, and quality control.

#### 2.4.2 Data cleaning and harmonization

Remediation and initial validation checks have identified missing data, inconsistencies, and discrepancies. Harmonization logic was developed and refined in line with institutional requirements and relevance. For each dataset, variables of interest (demographics information, tumor characteristics, treatment-related variables and biospecimen data) were compared across independent data sources, including EHRs, tumor registries reports, and LIMS. This step enabled the identification and analysis of discrepancy patterns across data sources, which serve as the foundation for defining the key challenges and proposed mitigation strategies described in this study.

#### 2.4.3 Quality control framework

The quality control process involves a multi-level, automated system of checks and validations. Automated validation checks were performed to evaluate completeness, internal validity, and temporal validity. Discrepant values were referred for evaluation. The final datasets were de-identified after the critical discrepancies were resolved.

## 3 Results

### 3.1 Overall data concordance

A comparison between tumor registries (TR) and Molecular Twin databases (R, REDCaP) against EHR-based datasets (CS-Link—EPIC™) showed very high concordance rates (>95%) for demographic variables such as sex, race, and ethnicity. This suggests that the systems can accurately record participants' demographic details, as shown in Table 1. On the other hand, there was a wide discordance in clinically essential factors. The TNM system and stage classification showed 72.8% concordance and 17% discordance, respectively. The dates of intervention, including the start dates of therapy and surgery, were no less variable (74.9% and 14.8%, respectively). The specificity of the diagnosis depended on how it was documented, which increased the challenges of harmonizing clinical records for longitudinal analysis. These results were aligned with the previous analysis of different cohorts (prostate and pancreas) shown in Supplementary Table S2.

**Table 1:** Evaluation of tumor registry data and molecular twin data database consistency against CS-link—EPIC™.

Variable	Source	Align (%)	Differences (%)	Missing (%)
Anatomical site	TR	93.7	–	6.3
	R	100	–	–
Diagnosis	TR	87.5	2.5	10.0
	R	84.9	15.1	–
Race	TR	91.1	2.6	6.3
	R	98.5	1.5	–
Sex	TR	93.7	–	6.3
	R	100	–	–
Ethnicity	TR	93.0	0.7	6.3
	R	100	–	–
Pathology available	R	95.9	4.1	–
Pathology case #	R	89.7	10.3	–
Date of diagnosis	TR	86.0	4.1	9.9
TNM (clinical code and stage group)	TR	72.8	17.0	10.2
Intervention dates	TR	74.9	14.8	10.3

TR, tumor registry data; R, molecular twin database (REDCap).

### 3.2 Sources of discordance

Asynchronous updates across systems and coding variations were among the factors contributing to data discordance. Also, while the EHRs reflected real-time clinical updates, tumor registry information was incorporated through scheduled curation cycles, resulting in occasional temporal discrepancies between systems. Additionally, free-text clinical documentation led to disparities that could not be identified in the structured fields.

### 3.3 Impact on cohort readiness

Variability was more pronounced in variables considered critical for cohort stratification analysis. Differences in treatment of milestone documentation influenced eligibility assessment and time-to-event analyses and required additional harmonization for molecular data integration.

### 3.4 Key challenges and proposed mitigation strategies

Review of the data management workflow identified areas where variability across data sources and update cadences introduced additional considerations for cohort readiness and downstream analyses. These observations highlight opportunities to further strengthen standardization and quality control practices. Table 2 summarizes the main areas identified and corresponding strategies to support improved standardization, data quality, and efficiency in future data integration efforts. Proposed measures include standardized data entry templates, structured documentation, automated cross-source validation, periodic audits, and selective use of Artificial Intelligence (AI)-supported data extraction to enhance the accuracy, consistency, and interoperability of longitudinal oncology datasets.

**Table 2:** Key challenges identified and proposed mitigation strategies in the molecular twin research umbrella data management workflow.

Challenge/Issue	Proposed mitigation strategies
Incomplete or inconsistent clinical and staging data	Standardized data entry templates, mandatory EHR fields, and AI-supported extraction from unstructured sources.
Heterogeneous clinical documentation	Structured documentation templates and terminology standards.
Data harmonization across sources	Automated cross-source validation, consistency checks, and periodic audits.
Diagnostic coding precision	Enforce ICD-10 coding standards through clinician training and periodic audits.
Asynchronous system updates	Centralized quality control (QC) pipeline and monitoring dashboards.
Manual data retrieval burden	Partially automating with AI-supported extraction, integration, and validation of data.

## 4 Discussion

This article highlights the dual nature of longitudinal patient-related data in oncology. While such data offer significant opportunities to advance precision medicine, they are inherently vulnerable to inaccuracies, inconsistencies, and biases that can compromise analytical outcomes (13, 15). These operational and structural challenges underscore the critical need for a centralized oncology database architecture capable of real-time updates and continuous quality control (16).

The four-step data management workflow described in this study aligns with the FAIR (Findable, Accessible, Interoperable, and Reusable) data principles. It does so by supporting a structured, transparent, and reproducible data integration process (21). The feasibility evaluation of the data request step contributes to findability and accessibility by standardizing data request procedures and ensuring controlled, yet traceable, access to data sources. The data cleaning and standardization step directly supports interoperability by transforming heterogeneous data from multiple databases into harmonized formats using consistent terminology and coding schemes (22). The proposed mitigation actions support the alignment of logic across sources. Finally, the data quality control and dataset release step reinforce reusability by ensuring data completeness, consistency, and validation, thereby increasing confidence in downstream analyses.

The concordance patterns found between Tumor Registry data and the Molecular Twin data, when matched with CS-Link—EPIC™, closely match those from previous EHR and Tumor Registry data. The data provide a concordance level of 95% for demographic information, 50%–95% for tumor data, and 10%–15% for missing data (17, 18). Lastly, the data points to the fact that, even with excellent concordance levels found in this analysis, there are still inconsistencies in data from human entry and in data with asynchronous updates. Although this study focuses on breast cancer cohorts within a single academic center, supplementary analyses conducted across additional Molecular Twin cohorts (pancreas and prostate), suggest that observed discordances in key clinical variables is not disease-specific but rather reflect broader data integration challenges (Supplementary Table S2).

The implementation of semi- or fully automated integration pipelines improves concordance and data completeness, as demonstrated by Langhout et al. (18), who reported 95%–100% accuracy in capturing registered cancer diagnoses compared with the Netherlands Cancer Registry.

The first challenge that emerged was dispersing data across various platforms and entities. As has been reported repeatedly in existing studies (23, 24), the lack of information on treatment, demographics, staging, and diagnosis can lead to bias and render any inference drawn from such data invalid. Therefore, it is essential to prioritize strategies, including the application of machine learning methods, to enhance data completeness, flag discrepancies and issue alerts for missing or incorrect data, extract data from unstructured text (e.g., pathology reports and encounter notes), and enable more robust analyses (23–25).

Specific data on clinical documentation consistency and the variability in structure, terminology, and detail, as well as on missing or incomplete information in research-essential cases (not clinical care), can lead to higher time load and an intensive workload for manual data retrieval, which impedes the accurate extraction of crucial clinical variables, including treatment, history, staging, and diagnosis, ultimately compromising data harmonization and downstream analysis. Addressing this challenge requires implementing structured clinical documentation templates, standardized terminology, and the use of ontology, in conjunction with continuous clinician training on the importance of comprehensive and uniform documentation (23, 25). As previously documented, structured documentation is associated with higher-quality records and reduced variability, leading to improved interoperability and greater potential for data reuse (22, 26, 27).

Data harmonization across sources posed a significant challenge that required substantial staff time from experts. This challenge arises from discrepancies across data sources. These discrepancies can lead to bias in how clinical outcomes are interpreted and in the level of confidence one has in those outcomes. To mitigate these effects, a verification procedure focused on data quality was implemented, in combination with automated data verification and validation, as well as manual data auditing. Moreover, the development of a computer-based verification system has improved the ability to detect incomplete or incorrect data in the records (28, 29).

Ajmal et al. (29) underscored the significance of inconsistent tumor classification codes in generating diagnostic coding heterogeneity, a phenomenon that limits diagnostic precision and reduces data interoperability. Addressing this challenge, standardized ICD-10 coding is essential, as it links EHR coding to its source. In our study, these standardization practices were introduced during the early phases of development, as part of a broader effort to improve data quality. Furthermore, the lack of standardized quality assurance and validation protocols significantly increases the risk of incorrect data interpretation, project timelines, and research productivity, and weakens the analysis (30). To address these issues, a centralized quality control pipeline within the database was established, which included fixed validation rules, automated error detection, and scheduled audits. Therefore, continuous data integrity monitoring became possible, allowing for early detection of anomalies and, by facilitating error reporting and dashboards, promoting transparency in maintaining data accuracy, integrity, and consistency (31, 32).

The process of data retrieval and its delivery required considerable manual work by specialized personnel, reflecting the complexity of compiling, reviewing, and harmonizing data across sources. The labor-intensive processes not only led to longer turnaround times but also increased the likelihood of delayed dataset delivery, thus potentially affecting project timelines and research productivity. To improve data workflow efficiency, partial automation of data extraction, compilation, and validation stages has been identified and implemented to overcome these challenges. The introduction of automation has been proven to significantly reduce human errors and lead to higher efficiency and easier, faster access to research-specific and curated datasets (33, 34).

In line with these efforts, ongoing initiatives within our data management workflow incorporate the emerging approaches, including the use of standardized, interoperable data models and FAIR-aligned infrastructures to improve cross-system data integration and machine readability, like OMOP data structure format DB (The Observational Medical Outcomes Partnership (OMOP) Common Data Model (CDM) (35). These efforts also include the application of AI-assisted methods for extracting and structuring data from heterogeneous clinical sources (36). In this context, emerging agentic (autonomous or semi-autonomous) AI paradigms that support data retrieval, structuring and validation offer a promising way to enhance efficiency while ensuring data quality through human oversight (37). More broadly, the integration of advanced computational approaches, including artificial intelligence and machine learning, represents a promising avenue to enhance the scalability, timeliness, and analytical value of oncology databases, enabling real-time data curation, automate data harmonization across sources, and facilitate rapid insights from complex datasets (18). However, the effectiveness and reliability of such tools are intrinsically dependent on the accuracy, completeness, and standardization of the input data.

Although this study does not aim to quantify the clinical impact of data inconsistencies or the effects of mitigation strategies, the systematic identification and resolution of discrepancies across various sources provides an indirect measure of the benefits associated with workflows that harmonize data in a structured manner. The quantitative improvements realized through the introduction of mitigation strategies are not fully assessed. However, the early introduction of a standardized requisition tracking form reduced error rate from paper form and LIMS, resulted in an over 95% concordance for biospecimen pre-analytical variables. The implementation of OMOP-like database decreases the turnaround time of cohort building from weeks to hours. Future studies should evaluate scalability to determine whether effectiveness varies across healthcare systems and institutional infrastructures.

The future role of cancer longitudinal collections (mainly utilizing biobanks) will entail a greater embrace of digital infrastructures that bridge the boundaries between clinical care, research, and biobanking (12). This digital transformation will not only refine the management of biospecimens and associated data but also the translation of discoveries into clinical benefit.

## 5 Conclusion

This paper unravels the complex and layered issues encountered in the administration of longitudinal oncology data and delineates the evolving role of modern biobanks, moving from well-annotated biospecimen repositories to more data-centric, integrated research platforms. The usefulness of this data in driving precision medicine heavily relies on the integrity, completeness, and interoperability of the data from different sources. The clinical annotation and infrastructure stages must be performed at a very high level, and, simultaneously, continuous education on clinical annotation and new technologies should be maintained so that the cohort conception is always ahead of potential future data needs and the cohort collected biospecimens are optimally utilized for high-end science. By employing sophisticated computational methods, primarily Artificial Intelligence and Machine Learning, these techniques have a significant transformational potential for real-time, adaptive, and scalable oncology databases. Nevertheless, it is only through continual investment in data infrastructure, validation frameworks, and clinician training that we will provide the opportunity to fully use these technologies and thus facilitate data-driven innovations in oncological research and clinical care. Such integrated frameworks, by converting fragmented and heterogeneous data into actionable knowledge, not only enable trust among participants and social accountability but also support translational research. Hence, the intersection of robust data infrastructure, advanced analytical methodologies, and patient-centered governance positions biobanks to serve not only as repositories of biospecimens but as dynamic engines driving translational research and the continuous improvement in clinical care.

The integration of longitudinal data in oncology poses several operational challenges. This case study will help the reader understand the key foundations for credible solutions in precision oncology, including high-quality data infrastructure, standardized procedures, quality control in centralized platforms, and medical guidance for automated procedures. Investments in quality in precision oncology solutions will play an even more critical role in laying the foundation for meaningful healthcare.

## Data Availability

Data access is not publicly available and requires approval by IRB and adherence to institutional data-sharing requirements. Requests to access these datasets should be submitted to Oncobiobank (oncobiobank@cshs.org).
