# Supplementary material for: Real-world evidence on data quality in precision oncology platforms: insights from the Molecular Twin Research Umbrella protocol
Source: Front Digit Health. 2026 Jun 30;8:1828544. doi: 10.3389/fdgth.2026.1828544 (PMC13365115; doi:10.3389/fdgth.2026.1828544)
Supplement: Supplementary file 1 [file Table1.docx]

**Supplementary Table 1**. Breast Cancer Data Model — Comprehensive Variable List.

| **Label** | **Level** | **Type** | **Description** | **Validation** |
| --- | --- | --- | --- | --- |
| **1. Patient Demographics** | | | | |
| PATIENT_ID | R | TEXT: The pseudonym may be a number | A pseudonym for the patient. The pseudonym must be generated in compliance with the pseudonyms of the cohort, and the Data Protection Policy's requirements may utilize a data source/biobank. | Generation of pseudonyms is the responsibility of the data source/biobank. Check for the non-speaking nature of the ID. |
| SEX | R | LIST_OF_VALUES [female; male; other] | Biological sex of the person, defined by chromosomes | One of permitted values |
| RACE_ETHNICITY | R | LIST_OF_VALUES [American Indian or Alaskan Native; Asian; Black or African American; White; One or more races; Not known] | Race and ethnicity of the participant. Label corrected from RACE _Ethnicity. | One of permitted values. |
| EDU_LEVEL | O | LIST_OF_VALUES [None; Graduated from high school or have high school equivalent; Some college; Completed vocational/technical training; Graduated from college; Some graduate/professional school; Completed graduate/professional school] | The education level of the participant | One of permitted values. |
| AGE_AT_PRIMARY_DIAGNOSIS | R | NATURAL_NUMBER [a] (0<=x) | Age at initial histopathological diagnosis (biopsy or surgical specimen of the primary tumor) rounded to years. | Non-negative integer. |
| AGE_AT_PRIMARY_RADIO_DIAGNOSIS | R | NATURAL_NUMBER [a] (0<=x) | Age at initial radiological diagnosis (X-Ray, CT, MRT, etc.) rounded to years. | Non-negative integer. |
| AGE_AT_PRIMARY_CLINICAL_DIAGNOSIS | R | NATURAL_NUMBER [a] (0<=x) | Age at initial clinical diagnosis (date of first investigation with suspected cancer) rounded to years. | Non-negative integer. |
| **2. Lifestyle and Risk Factors** | | | | |
| SMOKING_ANAMNESIS | R | LIST_OF_VALUES [yes former; yes current; no; not known] | Smoking history. If yes former or yes current => capture SMOKING_STARTED, SMOKING_STOPPED, SMOKING_PACKS_D. | Non-negative integer. |
| SMOKING_STARTED | O | DATE [] (ISO_8601_WITH_DAYS) | Date started to smoke | Year-Month-Day |
| SMOKING_STOPPED | O | DATE [] (ISO_8601_WITH_DAYS) | Date stopped to smoke | Year-Month-Day |
| SMOKING_YEARS | O | NATURAL_NUMBER [a] (0<=x) | Calculated: SMOKING_STOPPED minus SMOKING_STARTED; or if current, current date minus SMOKING_STARTED. | Non-negative integer. |
| SMOKING_PACKS_D | O | NATURAL_NUMBER [a] (0<=x) | Number of packs of cigarettes smoked per day. | Non-negative integer. |
| SMOKING_P_Y | O | NATURAL_NUMBER [a] (0<=x) | Pack-years smoking. Calculated as SMOKING_PACKS_D x SMOKING_YEARS. 1 P/Y = 1 pack/day x 1 year. | Non-negative integer. |
| VAPING_ANAMNESIS | R | LIST_OF_VALUES [yes former; yes current; no; not known] | History of vaping/e-cigarette use. | One of permitted values. |
| VAPING_FREQUENCY | O | LIST_OF_VALUES [daily, occasional] | Stratification of vaping/e-cigarette use. | One of permitted values. |
| VAPING_DURATION | O | NATURAL_NUMBER [a] (0<=x) | Duration of vaping/e-cigarette use in years. | Non-negative integer. |
| ALCOHOL_ANAMNESIS | R | LIST_OF_VALUES [yes former; yes current; no; not known] | History of alcohol use. | One of permitted values. |
| ALCOHOL_UNITS_WEEK | O | NATURAL_NUMBER [drinks/week] (0<=x) | Average number of standard alcoholic drinks per week. | Non-negative integer. |
| PSS_SCORE | O | NATURAL_NUMBER [a] (0<=x<40) | Perceived Stress Scale (PSS): 0-13 = low stress; 14-26 = moderate stress; 27-40 = high perceived stress. | Non-negative integer. |
| PHYS_ACTIVE | O | LIST_OF_VALUES [yes; no; don't know] | Participation in physical activities or exercises outside of regular job (e.g., running, walking, golf, gardening). | One of permitted values. |
| WEIGHT | O | NATURAL_NUMBER [a] (0<=x) | Weight in lb | Non-negative integer. |
| HEIGHT | O | NATURAL_NUMBER [a] (0<=x) | Height in inches | Non-negative integer. |
| BMI | O | NATURAL_NUMBER [a] (0<=x) | Calculated automatically: WEIGHT (lb) / [HEIGHT (in)]^2 x 703 | Non-negative integer. |
| SPEC_DIET | R | LIST_OF_VALUES [None; Mediterranean; Vegan; Vegetarian; Ketogenic; High-fat; Other; Not known] | Special diet followed by the patient. | One of permitted values. |
| **3. Reproductive and Gynecological History** | | | | |
| MENP_STATUS | R | LIST_OF_VALUES [yes; no; Unknown] | Menopausal status. If Yes => capture AGE_MENP. | One of permitted values |
| AGE_MENP | R | NATURAL_NUMBER [a] (0<=x) | Age at menopause rounded to years. | Non-negative integer. |
| AGE_MENARCHE | O | NATURAL_NUMBER [a] (9<=x<=20) | Age at menarche (first menstrual period) in years. | Non-negative integer. |
| GYN_HISTORY | O | LIST_OF_VALUES [none; LMP; gestation; hormone replacement use; OTC contraceptive; other] | Gynecological history relevant to breast cancer risk. If Yes => capture relevant sub-fields below. | One of permitted values. |
| AGE_FIRST_LIVE_BIRTH | O | NATURAL_NUMBER [a] (0<=x) | Age at first live birth in years. 0 = nulliparous. | Non-negative integer. |
| PARITY | O | NATURAL_NUMBER [a] (0<=x) | Number of live births. | Non-negative integer. |
| BREASTFEEDING | O | LIST_OF_VALUES [Yes; No; Unknown] | Whether the patient breastfed. If Yes, capture BREASTFEEDING_MONTHS. | One of permitted values. |
| BREASTFEEDING_MONTHS | O | NATURAL_NUMBER [month] (0<=x) | Total cumulative months of breastfeeding across all pregnancies. | Non-negative integer. |
| **4. Family and Comorbidity History** | | | | |
| CO_MORBID | R | LIST_OF_VALUES [Hypertension; Diabetes mellitus; Ischemic heart disease; Heart failure; Chronic kidney disease; COPD; Cerebrovascular disease; Peripheral vascular disease; Autoimmune disease; None; Other] | Comorbid conditions present at the time of diagnosis. | Non-empty values. |
| FAM_HISTORY | O | LIST_OF_VALUES [yes; no; Unknown] | Cancer history in the family. | One of permitted values. |
| FAM_HISTORY_DEGREE | O | LIST_OF_VALUES [First-degree; Second-degree; Both; Unknown] | Degree of relation of affected family member(s). First-degree = parent, sibling, child. | One of permitted values. |
| FAM_HISTORY_CANCER_TYPE | O | LIST_OF_VALUES [Breast; Ovarian; Both breast and ovarian; Pancreatic; Prostate (high-grade); Other; Unknown] | Type of cancer in affected family member(s). | One of permitted values. |
| **5. Clinical Study Participation** | | | | |
| CLINICAL_STUDY_PARTICIPANT | R | YES_NO [] (yes; no) | Participation in a clinical study. If Yes => capture CLINICAL_STUDY_ID. | One of permitted values. |
| CLINICAL_STUDY_ID | R | TEXT [] () | Clinical trial identifier (NCT number or equivalent). | Free text. |
| **6. Diagnosis and Imaging** | | | | |
| DATE_PRIM_DIAGNOSIS | R | DATE [] (ISO_8601_WITH_DAYS) | Date at which cancer was diagnosed for the first time. Histopathological diagnosis, obtained through biopsy or surgery, qualifies as a primary diagnosis. | Year-Month-Day |
| DATE_RADIO_DIAGNOSIS | R | DATE [] (ISO_8601_WITH_DAYS) | Date at which cancer was radiologically diagnosed for the first time. | Year-Month-Day |
| DATE_CLINIC_DIAGNOSIS | O | DATE [] (ISO_8601_WITH_DAYS) | Date of first clinical investigation with suspected cancer. | Year-Month-Day |
| METHOD_PRIMARY_RADIO_DIAGNOSIS | R | LIST_OF_VALUES [Mammography; X-Ray; CT; MRI; PET/CT; Other] | Primary radiological method used for initial diagnosis. Drives which DIAG_*_DONE field should be True. | One of permitted values. |
| DIAG_MAM_DONE | O | LIST_OF_VALUES [Done, data available; Done, data not available; Not done; Unknown] | Diagnostic mammography. Applies only if performed within the context of primary diagnosis. | One of permitted values |
| DIAG_LAST_MAM | R | LIST_OF_VALUES [1; 2; 3; 4; 5; 7; 9] | Time since last mammogram per 2021 BRFSS: 1=within past year; 2=1-2y; 3=2-3y; 4=3-5y; 5=5+ years; 7=don't know; 9=refused. | One of permitted values. |
| DIAG_BREAST_MRI_DONE | R | LIST_OF_VALUES [Done, data available; Done, data not available; Not done; Unknown] | Breast-specific MRI exam. Standard for high-risk screening, pre-operative extent-of-disease evaluation, and neoadjuvant response monitoring. | One of permitted values. |
| DIAG_CT_DONE | O | LIST_OF_VALUES [Done, data available; Done, data not available; Not done; Unknown] | Diagnostic CT exam. Applies only if performed within the context of primary diagnosis. | One of permitted values. |
| DIAG_X_RAY_DONE | O | LIST_OF_VALUES [Done, data available; Done, data not available; Not done; Unknown] | Diagnostic chest X-rays. Applies only if performed within the context of primary diagnosis. | One of permitted values. |
| DIAG_MRT_DONE | O | LIST_OF_VALUES [Done, data available; Done, data not available; Not done; Unknown] | Diagnostic MRI exam. Applies only if performed within the context of primary diagnosis. | One of permitted values. |
| DIAG_FULL_MRI_DONE | O | LIST_OF_VALUES [Done, data available; Done, data not available; Not done; Unknown] | Full-body MRI exam within the context of primary diagnosis. | One of permitted values. |
| DIAG_PET_DONE | O | LIST_OF_VALUES [Done, data available; Done, data not available; Not done; Unknown] | PET/CT diagnostic exam. Used for staging in locally advanced breast cancer (stage III) and suspected metastatic disease. Applies only within context of primary diagnosis. | One of permitted values. |
| DIAG_BIOPSY_TYPE | O | LIST_OF_VALUES [Core needle biopsy; Vacuum-assisted biopsy; Excisional biopsy; FNA; Other; Unknown] | Type of biopsy used to obtain histopathological diagnosis at primary diagnosis. | One of permitted values. |
| DIAG_CLIN_STAGE | O | LIST_OF_VALUES [cT0; cTis; cT1; cT2; cT3; cT4; cN0; cN1; cN2; cN3; cM0; cM1] | Clinical stage at primary diagnosis per AJCC 8th edition, based on clinical assessment prior to pathological confirmation. | One of permitted values. |
| **7. Pathology - Tumor Characteristics** | | | | |
| PATH_LATERALITY | O | LIST_OF_VALUES [Left; Right; Bilateral; Unknown] | Laterality of the primary breast tumor. | One of permitted values. |
| PATH_QUADRANT | O | LIST_OF_VALUES [UOQ; UIQ; LOQ; LIQ; Central; Nipple; Unknown] | Location of tumor within the breast. UOQ = upper outer quadrant, UIQ = upper inner, LOQ = lower outer, LIQ = lower inner. | One of permitted values. |
| PATH_MULTIFOCAL | O | LIST_OF_VALUES [Unifocal; Multifocal; Multicentric; Unknown] | Unifocal = single tumor; Multifocal = multiple foci within same quadrant or within 5 cm; Multicentric = foci in different quadrants or >5 cm apart. | One of permitted values. |
| PATH_DIAG_OVER | O | LIST_OF_VALUES [M; B] | Overall diagnostic classification: M = malignant, B = benign. | One of permitted values. |
| PATH_DCIS_COMPONENT | O | LIST_OF_VALUES [Present; Absent; DCIS only; Unknown] | Whether a DCIS (ductal carcinoma in situ) component is present alongside the invasive tumor. | One of permitted values. |
| PATH_TUMMOR_SIZE | O | NATURAL_NUMBER [mm] (0<=x) | Pathological tumor size in millimeters, largest invasive dimension. | Non-negative integer. |
| PATH_TUMOR_GRADE | R | LIST_OF_VALUES [G1; G2; G3; GX] | Histological grade. G1 = well differentiated; G2 = moderately differentiated; G3 = poorly differentiated; GX = cannot be assessed. | One of permitted values. |
| PATH_MOLECULAR_SUBTYPE | R | LIST_OF_VALUES [Luminal A; Luminal B (HER2-); Luminal B (HER2+); HER2-enriched; Triple Negative (TNBC); Unknown] | Breast cancer intrinsic molecular subtype derived from ER, PR, HER2, and Ki67. Luminal A: HR+/HER2-/Ki67<14%; Luminal B HER2-: HR+/HER2-/Ki67>=14%; Luminal B HER2+: HR+/HER2+; HER2-enriched: HR-/HER2+; TNBC: HR-/HER2-. Per St. Gallen 2023. | One of permitted values. |
| PATH_ER | O | LIST_OF_VALUES [Positive; Negative; Equivocal; Not done] | Estrogen receptor (ER) status by IHC. | One of permitted values. |
| PATH_PR | O | LIST_OF_VALUES [Positive; Negative; Equivocal; Not done] | Progesterone receptor (PR) status by IHC. | One of permitted values. |
| PATH_HER2 | O | LIST_OF_VALUES [Positive (3+); Equivocal (2+); Negative (0/1+); Not done] | HER2 receptor status by IHC and/or ISH. Equivocal (2+) requires reflex ISH testing. | One of permitted values. |
| PATH_AR | O | LIST_OF_VALUES [Positive; Negative; Not done] | Androgen receptor (AR) status by IHC. Positive defined as >=1% nuclear staining. | One of permitted values. |
| PATH_Ki67 | O | NATURAL_NUMBER [%] (0<=x<=100) | Ki67 proliferation index as percentage of positively stained tumor cells. Threshold: <14% low, 14-19% intermediate, >=20% high. | Non-negative integer. |
| PATH_TIL | O | NATURAL_NUMBER [%] (0<=x<=100) | Tumor-Infiltrating Lymphocytes (TIL) score as percentage of stromal area occupied by mononuclear inflammatory cells. | Non-negative integer. |
| PATH_LVI | O | LIST_OF_VALUES [Present; Absent; Indeterminate; Not assessed] | Lymph vascular invasion (LVI): presence of tumor emboli within lymphatic or blood vessel channels on H&E. | One of permitted values. |
| PATH_PNI | O | LIST_OF_VALUES [Present; Absent; Not assessed] | Perineural invasion (PNI): tumor cells within the perineural space. | One of permitted values. |
| PATH_P_53 | O | LIST_OF_VALUES [Mutant; Wild-type; Not done] | p53 protein expression status by IHC or TP53 mutation status. | One of permitted values. |
| **8. Pathology - Staging (Primary Diagnosis)** | | | | |
| PATH_T | R | LIST_OF_VALUES [TX; T0; Tis; T1; T2; T3; T4] | TX: primary tumor cannot be evaluated; T0: no evidence of primary tumor; Tis: carcinoma in situ; T1-T4: size and/or extent of primary tumor. | One of permitted values. |
| PATH_N | R | LIST_OF_VALUES [NX; N0; N1; N2; N3] | NX: regional lymph nodes cannot be evaluated; N0: no regional lymph node involvement; N1-N3: degree of regional lymph node involvement. | One of permitted values. |
| PATH_M | R | LIST_OF_VALUES [M0; M1] | M0: no distant metastasis; M1: distant metastasis present. | One of permitted values. |
| PATH_STAGE | R | LIST_OF_VALUES [0; I; II; III; IIIA; IIIB; IV] | Overall AJCC pathological stage derived from T, N, and M classification. | One of permitted values. |
| PATH_LYMPH_STATUS | R | LIST_OF_VALUES [Positive; Negative; Not done; Unknown] | Lymph node status. If Positive => capture PATH_NPL. | One of permitted values. |
| PATH_NPL | R | NATURAL_NUMBER [a] (0<=x) | Number of positive lymph nodes at primary surgery. | Non-negative integer. |
| COMP_PATH_STAGE_CLIN | O | LIST_OF_VALUES [Concordant; Upstaged; Downstaged; Not assessed] | Comparison between clinical (pre-operative) and pathological staging. Concordance or discordance between cTNM and pTNM staging. | One of permitted values. |
| **9. Genomics and Molecular Markers - Germline** | | | | |
| GEN_TEST_TYPE | O | LIST_OF_VALUES [Germline; Somatic; Both; Not tested] | Whether genetic testing was germline (blood/saliva), somatic (tumor tissue), or both. | One of permitted values. |
| GEN_PANEL_NAME | O | TEXT [] () | Name of the genetic testing panel used (e.g., Myriad myRisk, Foundation One CDx, Tempus xT). Affects which genes were assessed and comparability across cohorts. | Free text. |
| GEN_BRCA1 | O | LIST_OF_VALUES [Pathogenic variant; Likely pathogenic; VUS; Benign/Likely benign; Not tested] | Germline mutation status of BRCA1. Pathogenic variants associated with high lifetime risk of breast and ovarian cancer. | One of permitted values. |
| GEN_BRCA2 | O | LIST_OF_VALUES [Pathogenic variant; Likely pathogenic; VUS; Benign/Likely benign; Not tested] | Germline mutation status of BRCA2. Pathogenic variants associated with high lifetime risk of breast and ovarian cancer. | One of permitted values. |
| GEN_ATM | O | LIST_OF_VALUES [Pathogenic variant; Likely pathogenic; VUS; Benign/Likely benign; Not tested] | Germline mutation status of ATM. Heterozygous pathogenic variants linked to moderately elevated breast cancer risk. | One of permitted values. |
| GEN_PALB2 | O | LIST_OF_VALUES [Pathogenic variant; Likely pathogenic; VUS; Benign/Likely benign; Not tested] | Germline mutation status of PALB2. Pathogenic variants confer high breast cancer risk as a BRCA2 interactor. | One of permitted values. |
| GEN_TP53 | O | LIST_OF_VALUES [Pathogenic variant; Likely pathogenic; VUS; Benign/Likely benign; Not tested] | Germline mutation status of TP53. | One of permitted values. |
| GEN_CHEK2 | O | LIST_OF_VALUES [Pathogenic variant; Likely pathogenic; VUS; Benign/Likely benign; Not tested] | Germline mutation status of CHEK2. | One of permitted values. |
| GEN_PTEN | O | LIST_OF_VALUES [Pathogenic variant; Likely pathogenic; VUS; Benign/Likely benign; Not tested] | Germline mutation status of PTEN. | One of permitted values. |
| GEN_CDH1 | O | LIST_OF_VALUES [Pathogenic variant; Likely pathogenic; VUS; Benign/Likely benign; Not tested] | Germline mutation status of CDH1. | One of permitted values. |
| GEN_STK11 | O | LIST_OF_VALUES [Pathogenic variant; Likely pathogenic; VUS; Benign/Likely benign; Not tested] | Germline mutation status of STK11. | One of permitted values. |
| **10. Genomics and Molecular Markers - Somatic** | | | | |
| GEN_SOMATIC_PIK3CA | O | LIST_OF_VALUES [Mutated; Not mutated; Not tested] | Somatic PIK3CA mutation status. | One of permitted values. |
| GEN_SOMATIC_ESR1 | O | LIST_OF_VALUES [Mutated; Not mutated; Not tested] | Somatic ESR1 mutation status. | One of permitted values. |
| SOMATIC_BRCA_STATUS | O | LIST_OF_VALUES [Pathogenic variant; Likely pathogenic; VUS; Not mutated; Not tested] | Somatic (tumor) BRCA1/2 mutation status, distinct from germline testing. | One of permitted values. |
| **11. Prognostic Biomarker Assays** | | | | |
| LAB_ONCOTYPE_DX | O | LIST_OF_VALUES [LOW; INTERMEDIATE; HIGH] | Oncotype DX Recurrence Score risk group. Low: RS<18; Intermediate: RS 18-30; High: RS>30. | One of permitted values. |
| GEN_BCI | O | LIST_OF_VALUES [LOW; INTERMEDIATE; HIGH] | Breast Cancer Index (BCI) risk category. Low: BCI<5; Intermediate: 5<=BCI<6.4; High: BCI>=6.4. | One of permitted values. |
| LAB_MAMMAPRINT | O | LIST_OF_VALUES [Low risk; High risk; Not tested] | MammaPrint (Amsterdam 70-gene signature) result. | One of permitted values. |
| LAB_ENDOPREDICT | O | LIST_OF_VALUES [Low risk; High risk; Not tested] | EndoPredict (EPclin) score result. | One of permitted values. |
| SURV_RATES | O | NATURAL_NUMBER [%] (0<=x<=100) | Five-year relative survival rate at the population level for the relevant breast cancer subtype. Reported as percentage. | Non-negative integer (0-100). |
| **12. Surgery** | | | | |
| SURG_TYPE | R | LIST_OF_VALUES [Lumpectomy; Mastectomy; Nipple-sparing mastectomy; Skin-sparing mastectomy; Bilateral mastectomy; Other] | Type of surgical procedure performed on the primary breast tumor. | One of permitted values. |
| SURG_AXILLA_TYPE | O | LIST_OF_VALUES [SLNB dual-dye; SLNB radiotracer only; Full ALND; SLNB + ALND; None; Unknown] | Type of axillary surgery. SLNB = sentinel lymph node biopsy; ALND = axillary lymph node dissection. | One of permitted values. |
| SURG_MARGINS | O | LIST_OF_VALUES [R0; R1; R2; RX] | Surgical margin status. R0 = no tumor at margins; R1 = microscopic residual; R2 = macroscopic residual; RX = not assessable. | One of permitted values. |
| SURG_START_RELATIVE | R | NATURAL_NUMBER [week] (0<=x) | Time between initial diagnosis and surgery in weeks. | Non-negative integer. |
| SURG_RECONSTRUCTION | O | LIST_OF_VALUES [Immediate; Delayed; None; Unknown] | Breast reconstruction status and timing relative to mastectomy. | One of permitted values. |
| SURG_PCR_STATUS | O | LIST_OF_VALUES [pCR; non-pCR; Not applicable] | Pathological complete response (pCR) after neoadjuvant systemic therapy. pCR = no residual invasive disease in breast and axilla (ypT0/is ypN0). Not applicable if no neoadjuvant therapy was given. | One of permitted values. |
| SURG_SENTINEL_REMOVED | O | NATURAL_NUMBER [a] (0<=x) | Number of sentinel lymph nodes removed during SLNB. | Non-negative integer. |
| SURG_SENTINEL_POSITIVE | O | NATURAL_NUMBER [a] (0<=x) | Number of sentinel lymph nodes found positive for tumor. Must be <= SURG_SENTINEL_REMOVED. | Non-negative integer. |
| **13. Radiation Therapy** | | | | |
| RAD_DONE | R | LIST_OF_VALUES [Yes; No; Unknown] | Whether radiation therapy was administered as part of the treatment plan. | One of permitted values. |
| RAD_FIELDS | R | LIST_OF_VALUES [Breast only; Chest wall only; Breast + nodal; Chest wall + nodal; Other] | Radiation fields treated. Nodal fields include axillary, supraclavicular, and internal mammary lymph nodes. | One of permitted values. |
| RAD_BOOST_DONE | O | LIST_OF_VALUES [Yes; No; Unknown] | Whether a boost dose to the tumor bed was delivered after whole-breast irradiation. | One of permitted values. |
| RAD_HYPOFRACTIONATION | O | LIST_OF_VALUES [Conventional (25-28fx); Moderate hypofractionation (15-16fx); Ultra (FAST-FORWARD, 5fx); APBI; Unknown] | Radiation fractionation schedule | One of permitted values. |
| RAD_TOTAL_DOSE | O | NATURAL_NUMBER [Gy] (0<=x) | Total radiation dose administered in Gray (Gy). | Non-negative integer. |
| RAD_FRACTIONS | O | NATURAL_NUMBER [a] (0<=x) | Number of radiation fractions delivered. | Non-negative integer. |
| RAD_START_RELATIVE | R | NATURAL_NUMBER [week] (0<=x) | Start radiation therapy in weeks since initial diagnosis. | Non-negative integer. |
| RAD_END_RELATIVE | R | NATURAL_NUMBER [week] (0<=x) | End radiation therapy in weeks since initial diagnosis. | Non-negative integer. |
| RAD_CONCURRENT_SYS | O | LIST_OF_VALUES [None; Capecitabine; TDM1; Other] | Systemic therapy administered concurrently with radiation therapy. | One of permitted values. |
| RAD_DELAY_POST_SURG | O | NATURAL_NUMBER [week] (0<=x) | Delay between surgery and start of radiation therapy in weeks. | Non-negative integer. |
| **14. Systemic Therapy** | | | | |
| SYS_NEOADJUVANT_DONE | R | LIST_OF_VALUES [Yes; No; Unknown] | Whether any neoadjuvant (pre-operative) systemic therapy was administered. | One of permitted values. |
| SYS_CHEMO_DONE | R | LIST_OF_VALUES [Yes; No; Unknown] | Whether chemotherapy was administered (neoadjuvant or adjuvant). | One of permitted values. |
| SYS_CHEMO_SCHEME | R | LIST_OF_VALUES [AC; AC-T; AC-TH; TC; TCH; CMF; Dose-dense AC-T; Other] | Chemotherapy regimen. AC = doxorubicin + cyclophosphamide; T = taxane; H = trastuzumab; TC = taxane + cyclophosphamide. | One of permitted values. |
| SYS_CHEMO_SETTING | O | LIST_OF_VALUES [Neoadjuvant; Adjuvant; Both; Unknown] | Whether chemotherapy was given before (neoadjuvant) or after (adjuvant) surgery. | One of permitted values. |
| SYS_ENDOCRINE_DONE | R | LIST_OF_VALUES [Yes; No; Unknown] | Whether endocrine (hormone) therapy was administered. | One of permitted values. |
| SYS_ENDOCRINE_AGENT | O | LIST_OF_VALUES [Tamoxifen; Aromatase inhibitor; LHRH agonist; Fulvestrant; CDK4/6 inhibitor + AI; Other] | Endocrine therapy agent(s) used. CDK4/6 inhibitors may be combined with AI. | One of permitted values. |
| SYS_ENDOCRINE_DURATION | O | NATURAL_NUMBER [month] (0<=x) | Duration of endocrine therapy in months. | Non-negative integer. |
| SYS_TARGETED_DONE | R | LIST_OF_VALUES [Yes; No; Unknown] | Whether targeted therapy (non-ADC, non-immunotherapy) was administered. | One of permitted values. |
| SYS_TARGETED_AGENT | O | LIST_OF_VALUES [Trastuzumab; Pertuzumab; Neratinib; Lapatinib; Olaparib; Talazoparib; Other] | Targeted therapy agent(s) used. Olaparib/Talazoparib (PARP inhibitors) included here for pre-metastatic settings. | One of permitted values. |
| SYS_ADC_DONE | R | LIST_OF_VALUES [Yes; No; Unknown] | Whether an antibody-drug conjugate (ADC) was administered. | One of permitted values. |
| SYS_ADC_AGENT | O | LIST_OF_VALUES [T-DM1 (trastuzumab emtansine); T-DXd (trastuzumab deruxtecan); Sacituzumab govitecan; Datopotamab deruxtecan; Other] | ADC agent(s) used. | One of permitted values. |
| SYS_IMMUNOTHERAPY_DONE | R | LIST_OF_VALUES [Yes; No; Unknown] | Whether immune checkpoint inhibitor therapy was administered. | One of permitted values. |
| SYS_IMMUNOTHERAPY_AGENT | O | LIST_OF_VALUES [Pembrolizumab; Atezolizumab; Other; Unknown] | Immune checkpoint inhibitor agent(s) used. Populate if SYS_IMMUNOTHERAPY_DONE = Yes. | One of permitted values. |
| SYS_BISPHOSPHONATE | O | LIST_OF_VALUES [Yes; No; Unknown] | Whether adjuvant bisphosphonate therapy was administered (e.g., zoledronic acid, clodronate). | One of permitted values. |
| SYS_OLAPARIB_DONE | O | LIST_OF_VALUES [Yes; No; Unknown] | Whether olaparib (PARP inhibitor) was administered in the adjuvant setting. | One of permitted values. |
| SYS_START_RELATIVE | R | NATURAL_NUMBER [week] (0<=x) | Start of systemic therapy in weeks since initial diagnosis. | Non-negative integer. |
| SYS_END_RELATIVE | R | NATURAL_NUMBER [week] (0<=x) | End of systemic therapy in weeks since initial diagnosis. | Non-negative integer. |
| **15. Therapy Response (Neoadjuvant / Primary)** | | | | |
| NADRJ_RECIST_RESPONSE | O | LIST_OF_VALUES [Complete response; Partial response; Stable disease; Progressive disease] | Best overall response per RECIST 1.1 criteria at primary/neoadjuvant assessment. | One of permitted values. |
| **16. Adverse Events** | | | | |
| AE_GRADE_MAX | O | LIST_OF_VALUES [1; 2; 3; 4; 5] | Maximum CTCAE grade of any adverse event recorded during treatment. | One of permitted values. |
| AE_NEUROPATHY | O | LIST_OF_VALUES [Grade 0; Grade 1; Grade 2; Grade 3; Grade 4] | Maximum grade of chemotherapy-induced peripheral neuropathy (CIPN). | One of permitted values. |
| AE_CARDIOTOXICITY | O | LIST_OF_VALUES [Yes; No; Unknown] | Whether cardiotoxicity was documented. Includes LVEF decline (>=10% absolute drop to <53%), clinical heart failure. | One of permitted values. |
| AE_LVEF_BASELINE | O | NATURAL_NUMBER [%] (0<=x<=100) | Left ventricular ejection fraction (LVEF) at baseline before cardiotoxic therapy (echocardiogram or MUGA scan). | Non-negative integer 0-100. |
| AE_LVEF_NADIR | O | NATURAL_NUMBER [%] (0<=x<=100) | Lowest recorded LVEF during or after treatment. Populate if AE_CARDIOTOXICITY = Yes. | Non-negative integer 0-100. |
| AESI_IMMUNE_ENDO | O | LIST_OF_VALUES [Yes; No; Unknown] | Immune-related adverse event: endocrinopathy (e.g., thyroiditis, adrenal insufficiency, hypophysitis). | One of permitted values. |
| AESI_PNEUMONITIS | O | LIST_OF_VALUES [Yes; No; Unknown] | Immune-related adverse event: pneumonitis. | One of permitted values. |
| AESI_NEPHRITIS | O | LIST_OF_VALUES [Yes; No; Unknown] | Immune-related adverse event: nephritis. | One of permitted values. |
| AESI_HEPATITIS | O | LIST_OF_VALUES [Yes; No; Unknown] | Immune-related adverse event: hepatitis (irAE). | One of permitted values. |
| AESI_LFT_ELEVATED | O | LIST_OF_VALUES [Yes; No; Unknown] | Liver function test elevation (ALT/AST > ULN). | One of permitted values. |
| **17. Metastatic and Recurrent Disease** | | | | |
| TIME_OF_RECURRENCE_RELATIVE | R | NATURAL_NUMBER [week] (0<=x) | Weeks between primary diagnosis and first diagnosed recurrence. Weeks = months x 4 if only months are available. | Non-negative integer. |
| MET_DATE_FIRST_RECURRENCE | R | DATE [] (ISO_8601_WITH_DAYS) | Absolute date of first documented recurrence or distant metastasis. | Year-Month-Day |
| MET_RECURRENCE_TYPE | O | LIST_OF_VALUES [Local (ipsilateral breast); Regional (lymph nodes); Contralateral breast; Distant; Combined local+distant; Unknown] | Type of first recurrence event. Distinguishes local/regional recurrence from distant metastasis. | One of permitted values. |
| MET_SITE | R | LIST_OF_VALUES [Bone; Lung; Liver; Brain; Lymph nodes; Skin; Adrenal; Peritoneum; Other] | Site(s) of distant metastasis. | Multiple selections permitted. |
| PATH_STAGE_AT_RECURRENCE | O | LIST_OF_VALUES [0; I; II; III; IIIA; IIIB; IV] | Overall AJCC stage at time of recurrence. | One of permitted values. |
| PATH_T_AT_RECURRENCE | O | LIST_OF_VALUES [TX; T0; Tis; T1; T2; T3; T4] | Primary tumor T classification at time of recurrence. | One of permitted values. |
| PATH_N_AT_RECURRENCE | O | LIST_OF_VALUES [NX; N0; N1; N2; N3] | Regional lymph node N classification at time of recurrence. | One of permitted values. |
| PATH_M_AT_RECURRENCE | O | LIST_OF_VALUES [M0; M1] | Distant metastasis M classification at time of recurrence. | One of permitted values. |
| MET_LINES_OF_THERAPY | O | NATURAL_NUMBER [a] (0<=x) | Number of lines of systemic therapy received for metastatic or recurrent disease. | Non-negative integer. |
| MET_GENOMICS_PDL1 | O | LIST_OF_VALUES [Positive; Negative; Not tested] | PD-L1 expression status at time of metastatic disease evaluation (e.g., for immunotherapy eligibility). | One of permitted values. |
| MET_TMB | O | NATURAL_NUMBER [mut/Mb] (0<=x) | Tumor mutational burden (TMB) at time of metastatic evaluation. Expressed as mutations per megabase. | Non-negative integer. |
| MET_RECIST_RESPONSE | O | LIST_OF_VALUES [Complete response; Partial response; Stable disease; Progressive disease] | Best overall response per RECIST 1.1 for each line of metastatic therapy. For primary/neoadjuvant response, see NADRJ_RECIST_RESPONSE (section 15). | One of permitted values. |
| PFS_LINE1_RELATIVE | O | NATURAL_NUMBER [week] (0<=x) | Progression-free survival (PFS) for first line of metastatic therapy in weeks from start of that therapy. | Non-negative integer. |
| **18. Vital Status and Survival Endpoints** | | | | |
| VITAL_STATUS | R | LIST_OF_VALUES [death due to cancer; death due to other reasons; death for unknown reasons; person is still alive; unknown] | Vital status of the patient. | One of permitted values. |
| VITAL_STATUS_TIMESTAMP | O | DATE [] (ISO_8601_WITH_DAYS) | Timestamp of last update of vital status. Required if vital status is provided, otherwise optional. | Year-Month-Day |
| OS_TIME_RELATIVE | O | NATURAL_NUMBER [week] (0<=x) | Overall Survival (OS): time from initial diagnosis to death or last follow-up in weeks. | Non-negative integer. |
| IDFS_STATUS | O | LIST_OF_VALUES [Event; No event; Unknown] | Invasive Disease-Free Survival (IDFS) status. Event = ipsilateral invasive breast recurrence, contralateral invasive breast cancer, distant recurrence, or death from any cause. | One of permitted values. |
| IDFS_TIME_RELATIVE | O | NATURAL_NUMBER [week] (0<=x) | Time for IDFS event or last follow-up in weeks since initial diagnosis. | Non-negative integer. |
| DDFS_STATUS | O | LIST_OF_VALUES [Event; No event; Unknown] | Distant Disease-Free Survival (DDFS) status. Event = first distant recurrence or death from any cause. | One of permitted values. |
| DDFS_TIME_RELATIVE | O | NATURAL_NUMBER [week] (0<=x) | Time for DDFS event or last follow-up in weeks since initial diagnosis. | Non-negative integer. |
| LRFS_STATUS | O | LIST_OF_VALUES [Event; No event; Unknown] | Local Recurrence-Free Survival (LRFS) status. Event = ipsilateral breast tumor recurrence (IBTR) only. | One of permitted values. |
| LRFS_TIME_RELATIVE | O | NATURAL_NUMBER [week] (0<=x) | Time to local recurrence event or last follow-up in weeks since initial diagnosis. | Non-negative integer. |
| **19. Biobank Samples** | | | | |
| BB_SAMP_TISSUE_FFPE | R | LIST_OF_VALUES [Available; Not available; Unknown] | Availability of formalin-fixed paraffin-embedded (FFPE) tumor tissue block in the biobank. | One of permitted values. |
| BB_SAMP_TISSUE_FROZEN | R | LIST_OF_VALUES [Available; Not available; Unknown] | Availability of snap-frozen tumor tissue specimen in the biobank. | One of permitted values. |
| BB_SAMP_EDTA_WH_BLOOD | O | LIST_OF_VALUES [Available; Not available; Unknown] | Availability of EDTA whole blood specimen in the biobank. | One of permitted values. |
| BB_SAMP_EDTA_BUFFY | O | LIST_OF_VALUES [Available; Not available; Unknown] | Availability of buffy coat (leukocyte layer) from EDTA tube in the biobank. | One of permitted values. |
| BB_SAMP_EDTA_PLASMA | O | LIST_OF_VALUES [Available; Not available; Unknown] | Availability of plasma separated from EDTA whole blood. | One of permitted values. |
| BB_SAMP_SERUM | O | LIST_OF_VALUES [Available; Not available; Unknown] | Availability of serum specimen in the biobank. | One of permitted values. |
| BB_SAMP_LIHEP_PLASMA | O | LIST_OF_VALUES [Available; Not available; Unknown] | Availability of plasma separated from lithium heparin tube. | One of permitted values. |
| BB_SAMP_CITR_PLASMA | O | LIST_OF_VALUES [Available; Not available; Unknown] | Availability of plasma separated from citrate tube. | One of permitted values. |
| BB_SAMP_CTDNA | O | LIST_OF_VALUES [Available; Not available; Unknown] | Availability of circulating tumor DNA (ctDNA) / liquid biopsy. | One of permitted values. |
| BB_SAMP_URINE | O | LIST_OF_VALUES [Available; Not available; Unknown] | Availability of urine specimen for metabolomics, hormonal, or toxicological analysis. | One of permitted values. |
| BB_SAMP_STOOL_MICROBIOM | O | LIST_OF_VALUES [Available; Not available; Unknown] | Availability of stool sample for microbiome analysis. | One of permitted values. |
| BB_SAMP_SALIVA_MICROBIOM | O | LIST_OF_VALUES [Available; Not available; Unknown] | Availability of saliva sample for microbiome analysis. | One of permitted values. |
| BB_SAMP_HAIR | O | LIST_OF_VALUES [Available; Not available; Unknown] | Availability of hair specimen (e.g., for hormonal or toxicological analysis). | One of permitted values. |
| BB_SAMP_COLLECTION_DATE | R | DATE [] (ISO_8601_WITH_DAYS) | Timestamp of sample collection. | Year-Month-Day |
| SAMPLE_ID | R | TEXT [] () | Unique identifier within the Biobank. | One of permitted values. |

Abbreviations: R = Required; O = Optional; VUS = Variant of Uncertain Significance; IHC = Immunohistochemistry; ISH = In Situ Hybridisation; HR = Hormone Receptor; TNBC = Triple Negative Breast Cancer; SLNB = Sentinel Lymph Node Biopsy; ALND = Axillary Lymph Node Dissection; ADC = Antibody-Drug Conjugate; pCR = Pathological Complete Response; IDFS = Invasive Disease-Free Survival; DDFS = Distant Disease-Free Survival; LRFS = Local Recurrence-Free Survival; OS = Overall Survival.
