# Supplementary material for: Real-world evidence on data quality in precision oncology platforms: insights from the Molecular Twin Research Umbrella protocol
Source: Front Digit Health. 2026 Jun 30;8:1828544. doi: 10.3389/fdgth.2026.1828544 (PMC13365115; doi:10.3389/fdgth.2026.1828544)
Supplement: Supplementary file 2 [file Table2.docx]

**Supplementary Table 2.**  Evaluation of Tumor Registry data and Molecular Twin Data Database consistency against CS-Link – EPIC^TM^ for Pancreas and Prostate Cohorts.

| **Variable / Anatomical Site** | **Source** | **Pancreas** | | | **Prostate** | | |
| --- | --- | --- | --- | --- | --- | --- | --- |
|  |  | **Align (%)** | **Differences (%)** | **Missing (%)** | **Align (%)** | **Differences (%)** | **Missing (%)** |
| Anatomical Site | TR | 93.87 | 2.36 | 3.77 | 91.16 | 2.33 | 6.51 |
|  | R | 100 | – | – | 100 | – | – |
| Diagnosis | TR | 83.02 | 9.43 | 7.55 | 82.79 | 10.23 | 6.98 |
|  | R | 83.96 | 16.04 | – | 94.42 | 5.58 | – |
| Race | TR | 90.57 | 6.13 | 3.3 | 89.77 | 3.72 | 6.51 |
|  | R | 99.06 | 0.94 | – | 99.53 | 0.47 | – |
| Gender | TR | 96.7 | – | 3.3 | 93.49 | – | 6.51 |
|  | R | 100 | – | – | 100 | – | – |
| Ethnicity | TR | 93.87 | 2.83 | 3.3 | 92.09 | 1.4 | 6.51 |
|  | R | 99.06 | 0.94 | – | 100 | – | – |
| Pathology Available | R | 80.72 | 19.28 | – | 77.79 | 22.21 | – |
| Pathology Case # | R | 81.48 | 18.52 | – | 82.86 | 17.14 | – |
| Date of Diagnosis | TR | 84.43 | 8.49 | 7.08 | 78.14 | 15.35 | 6.51 |
| TNM (Clinical Code and Stage Group) | TR | 78.6 | 13.75 | 7.65 | 80.93 | 11.61 | 7.46 |
| Intervention Dates | TR | 89.62 | 2.36 | 8.02 | 82.09 | 9.77 | 8.14 |

Abbreviations**:** TR, Tumor Registry data; R, Molecular Twin Database (REDCap)
